# The importance of mental and sexual health in addressing people with hiv - the double stigma

**DOI:** 10.1192/j.eurpsy.2021.1469

**Published:** 2021-08-13

**Authors:** S. Nascimento, M. Gonçalves

**Affiliations:** Psychiatry, Centro hospitalar psiquiátrico de lisboa, Lisboa, Portugal

**Keywords:** Preexposure prophylaxis, HIV, Mental health problems, sexual health

## Abstract

**Introduction:**

Having a mental disorder is associated with increased vulnerability to the transmission of the Human Immunodeficiency Virus (HIV) and the prevalence of HIV is higher in people with a severe mental disorder. People with psychiatric comorbidities such as bipolar affective disorder and depressive disorder, post-traumatic stress disorder (physical or sexual abuse) and/or psychoactive substance use have a higher risk of HIV infection.

**Objectives:**

This work is intended to expose the importance of integrating mental health care with the care of HIV patients.

**Methods:**

The authors conducted a non-systematic review of the literature, conducting research through Pubmed and Medscape using the keywords ‘Preexposure prophylaxis’, ‘HIV’, ‘Mental health problems’.

**Results:**

Several factors may contribute to the high comorbidity between HIV and Mental Disorders, including socio-demographic factors, weak social and environmental structures, as well as internalized stigma, social and experienced discrimination. Mental health problems may interfere with the care needed for prevention, including regular HIV testing and/or adherence to Preexposure Prophylaxis (PrEP); and influence access to and adherence to antiretroviral treatment.

**Conclusions:**

This compelling evidence makes the necessary contribution of integrating mental health into an assessment and continuous treatment of the HIV patient, on the other hand, the assessment and treatment of mental disorders should address sexual health.

